# DetEdit: A graphical user interface for annotating and editing events detected in long-term acoustic monitoring data

**DOI:** 10.1371/journal.pcbi.1007598

**Published:** 2020-01-13

**Authors:** Alba Solsona-Berga, Kaitlin E. Frasier, Simone Baumann-Pickering, Sean M. Wiggins, John A. Hildebrand

**Affiliations:** Scripps Institution of Oceanography, University of California, San Diego, La Jolla, California, United States of America; Hebrew University of Jerusalem, ISRAEL

## Abstract

Passive acoustic monitoring has become an important data collection method, yielding massive datasets replete with biological, environmental and anthropogenic information. Automated signal detectors and classifiers are needed to identify events within these datasets, such as the presence of species-specific sounds or anthropogenic noise. These automated methods, however, are rarely a complete substitute for expert analyst review. The ability to visualize and annotate acoustic events efficiently can enhance scientific insights from large, previously intractable datasets. A MATLAB-based graphical user interface, called *DetEdit*, was developed to accelerate the editing and annotating of automated detections from extensive acoustic datasets. This tool is highly-configurable and multipurpose, with uses ranging from annotation and classification of individual signals or signal-clusters and evaluation of signal properties, to identification of false detections and false positive rate estimation. *DetEdit* allows users to step through acoustic events, displaying a range of signal features, including time series of received levels, long-term spectral averages, time intervals between detections, and scatter plots of peak frequency, RMS, and peak-to-peak received levels. Additionally, it displays either individual, or averaged sound pressure waveforms, and power spectra within each acoustic event. These views simultaneously provide analysts with signal-level detail and encounter-level context. *DetEdit* creates datasets of signal labels for further analyses, such as training classifiers and quantifying occurrence, abundances, or trends. Although designed for evaluating underwater-recorded odontocete echolocation click detections, *DetEdit* can be adapted to almost any stereotyped impulsive signal. Our software package complements available tools for the bioacoustic community and is provided open source at https://github.com/MarineBioAcousticsRC/DetEdit.

This is a *PLOS Computational Biology* Software paper.

## Introduction

A variety of animals produce species-specific acoustic signals, including marine mammals, fish, crustaceans, primates, bats, birds, anurans, and insects [1–9]. Acoustic analysis has become a standard method in studies of animal vocal communication, and manual detection of acoustic cues was initially the common practice. However, advances in recording hardware speeds, battery life and data storage capacity have increased the rate of acoustic data accumulation to a point where reliance on manual analysis has become unmanageable. This is the case for bottom-mounted acoustic recorders that record 2-byte samples at 200,000 samples/sec autonomously for months to over a year, yielding up to 16 terabytes of acoustic data storage from a single deployment. Automated detection and classification algorithms have become necessary for the analysis process. These algorithms provide more consistent and comparable estimates throughout a study period and across studies when processing long-term time series. They are less prone to bias than human analysts, and can be quantified more objectively. However, they cannot be used without supervision, and typically require performance evaluation or correction at some point in the processing pipeline. For instance, a detection algorithm’s performance must be estimated across a representative range of recording conditions. Labels may need to be applied to detections to train automatic classification routines, and misclassifications may need to be quantified or corrected [10]. In the absence of tools capable of performing these tasks across large datasets, the tasks are often done manually by an analyst sampling a subset of data which may not be representative of the full dataset. Further, because they are time consuming to estimate, performance metrics are often only measured for a few datasets and then assumed to apply more broadly. Despite the potential of automated classification tools to produce reliable, repeatable decisions and results from large datasets, manual review remains an important part of the process for additional scientific insights, since analysts are best able to judge the context-dependent nature of biological data.

The aim of our software described herein is to facilitate efficient exploration, characterization, and correction of signal detections and classifications in large, long-term acoustic datasets. A custom graphical user interface (GUI) tool, *DetEdit*, is presented to accelerate and enhance the process of acoustic big data analysis by combining signal-level detail with encounter-level context. This tool will facilitate training of machine learning algorithms for species classification. It can be applied to stereotyped signals that are characterized by spectral shape, such as underwater sounds produced by odontocetes, crustaceans, sonar, and ships, or terrestrial sounds, including calls made by bats and swiftlets. As examples, the use of *DetEdit* is illustrated with two case studies of odontocetes from long-term time series.

## Design and implementation

### Overview

**Fig 1:**
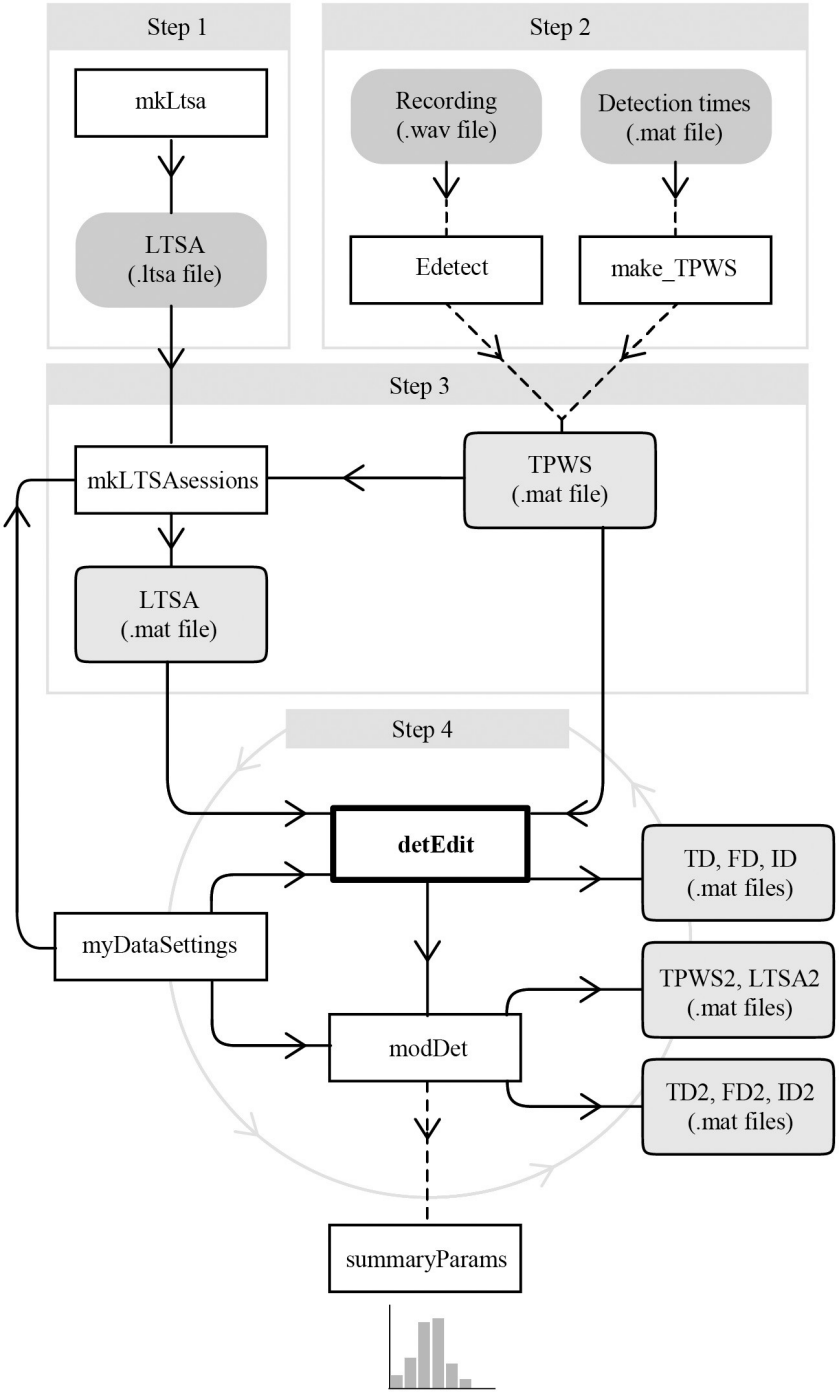
Schematic representation of the *DetEdit* workflow. The workflow is a multi-step process where the user: (1) creates a Long Term Spectral Average (LTSA, see Workflow section for details) of the complete long-term acoustic recording, (2) provides detections to create a TPWS (start Time, Peak-to-peak received sound pressure level, Waveform, Spectrum) file or executes a simple energy detector, (3) creates LTSAs per bout, (4) executes the interface *DetEdit* to annotate detections, and (5) identify false positive detections. If desired, histograms and plots are created to summarize the annotated data. The schematic workflow includes functions (white boxes), data files (gray boxes), and optional steps (dashed lines).

The *DetEdit* package provides a set of tools designed to parse, manipulate, and visualize acoustic detections in a workflow format using a user-interface developed in MATLAB (Mathworks, Natick, MA). The package, which depends on the core detEdit function, implements a hierarchical pipeline that incorporates data preprocessing, visualization, and manipulation tools (Fig 1). The main functions and files found in the repository with a brief summary of their usage is described at https://github.com/MarineBioAcousticsRC/DetEdit/wiki/How-It-Works#Table. The processing pipeline begins by using *DetEdit* to navigate through successive encounters, with modifications made to annotation matrices based on user decisions. This process is repeated as needed and also supports manual assessment of false positive rates.

A set of pre-processing tools are provided to produce the input required to use detEdit, the central user-interface tool. A generic impulse detector (Edetect) is provided to detect signals and produce matrices of detection parameters for input into *DetEdit*. Others (mkLtsa, mkLTSAsessions), compute spectral averages. The primary tool, detEdit, allows users to visualize the data with specific acoustic features, interactively explore and manually annotate data. Other functions, including modDet and summaryParams are post-processing tools, manipulating detections based on user annotations and producing plot-based data summaries.

### Workflow

The process of editing detections involves following several steps to create the parameters needed for the interface (Fig 1):

**Step 1: Create LTSA files**. The *DetEdit* package relies on mkLtsa to read audio files (wav format) and compress these data into long-term spectral averages (LTSA), which are power spectra calculated at regular intervals for the entire audio file [11]. LTSA are produced for the audio files by specifying the time average and frequency-bin size. They facilitate exploration and provide an easily visualized overview for long-term acoustic data.

**Step 2: Create TPWS files**. The input to the *DetEdit* GUI is a MATLAB binary file labeled as “TPWS” (Time, Peak-to-peak received levels, Waveform and Spectra parameters) that contains matrices of the acoustic detection parameters. These matrices can be created manually by the user or with make_TPWS which builds the following four primary variables to visualize detections in the interface:

- MTT: a vector of detection times.
- MPP: a vector of peak-to-peak received levels.
- MSP: a matrix of normalized spectra.
- MSN: a matrix of normalized waveforms.

If no detections are given, the package provides Edetect to assist in detecting acoustic events in audio files. This generic detector applies a configurable band pass filter and returns events that meet or exceed a minimum received level threshold and satisfy other configurable acoustic criteria.

**Step 3: Create LTSA Sessions files**. Detections are grouped in user-defined time bouts, defined as periods of stereotyped signals separated from prior and subsequent detections by a minimum specified time gap. mkLTSAsessions takes an LTSA and produces the following two variables needed to represent subsets of the LTSA per bout:

- pt: a vector of power spectral average start times.
- pwr: a matrix of power spectral density averages.

#### Visualization, annotation, and manipulation of data

**Fig 2:**
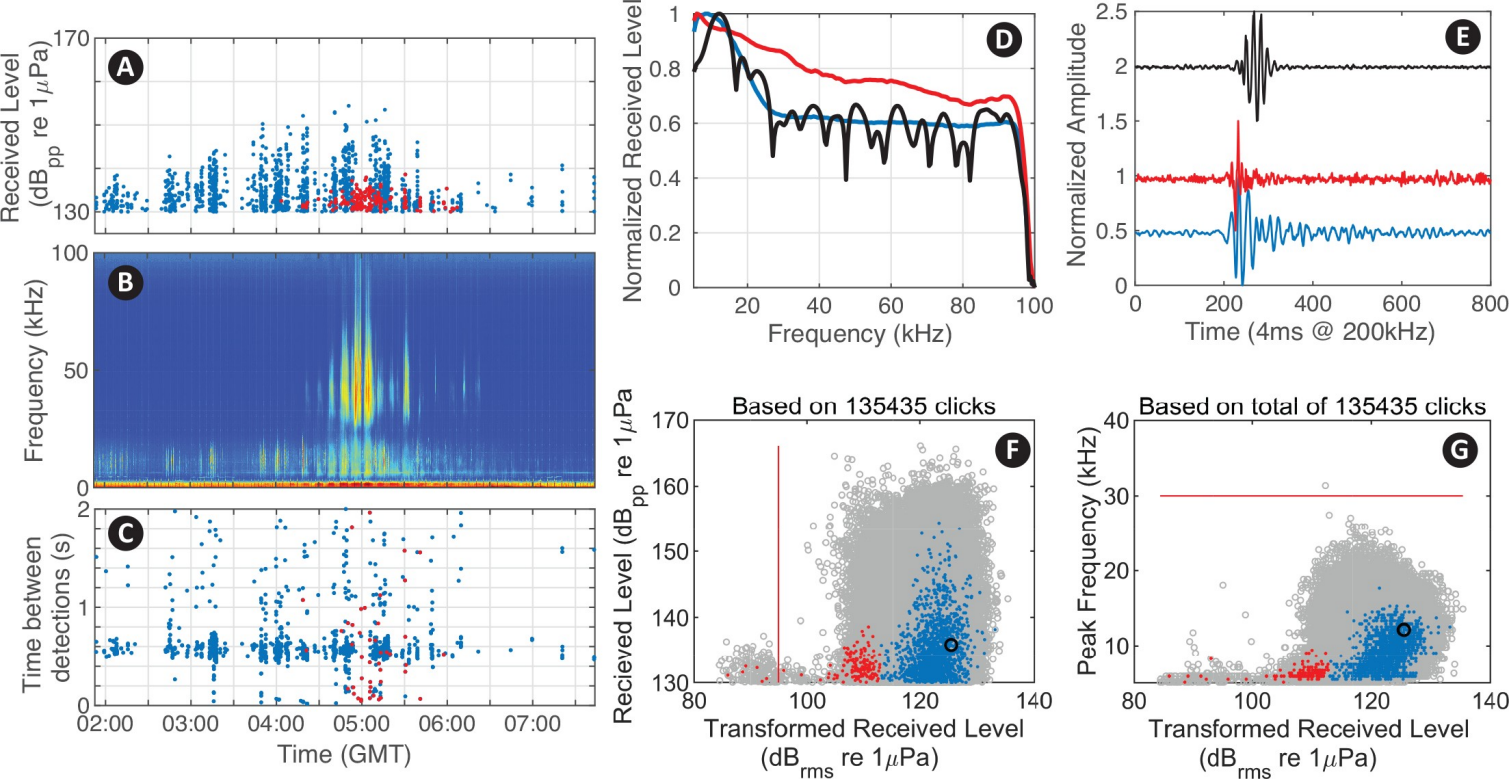
Visualization of acoustic data in the *DetEdit* interface. Seven panels are displayed with three over the event period: (A) RL_pp_, (B) LTSA, (C) time interval between detections; and four other showing various detection metrics and details: (D) normalized spectral density, (E) normalized waveforms, (F) RL_pp_ versus transformed RL_rms_, and (G) peak frequencies versus transformed RL_rms_ for sperm whale (*Physeter macrocephalus*) signal detections with true detections as blue, manually identified false detections of delphinid signals as red, and one detection using the selection tool displayed as black. All detections from the recording are shown in gray on the background if specified by the user to ease comparison of distributions across bouts. Customized classification thresholds are displayed as thin red lines (F and G).

**Fig 3:**
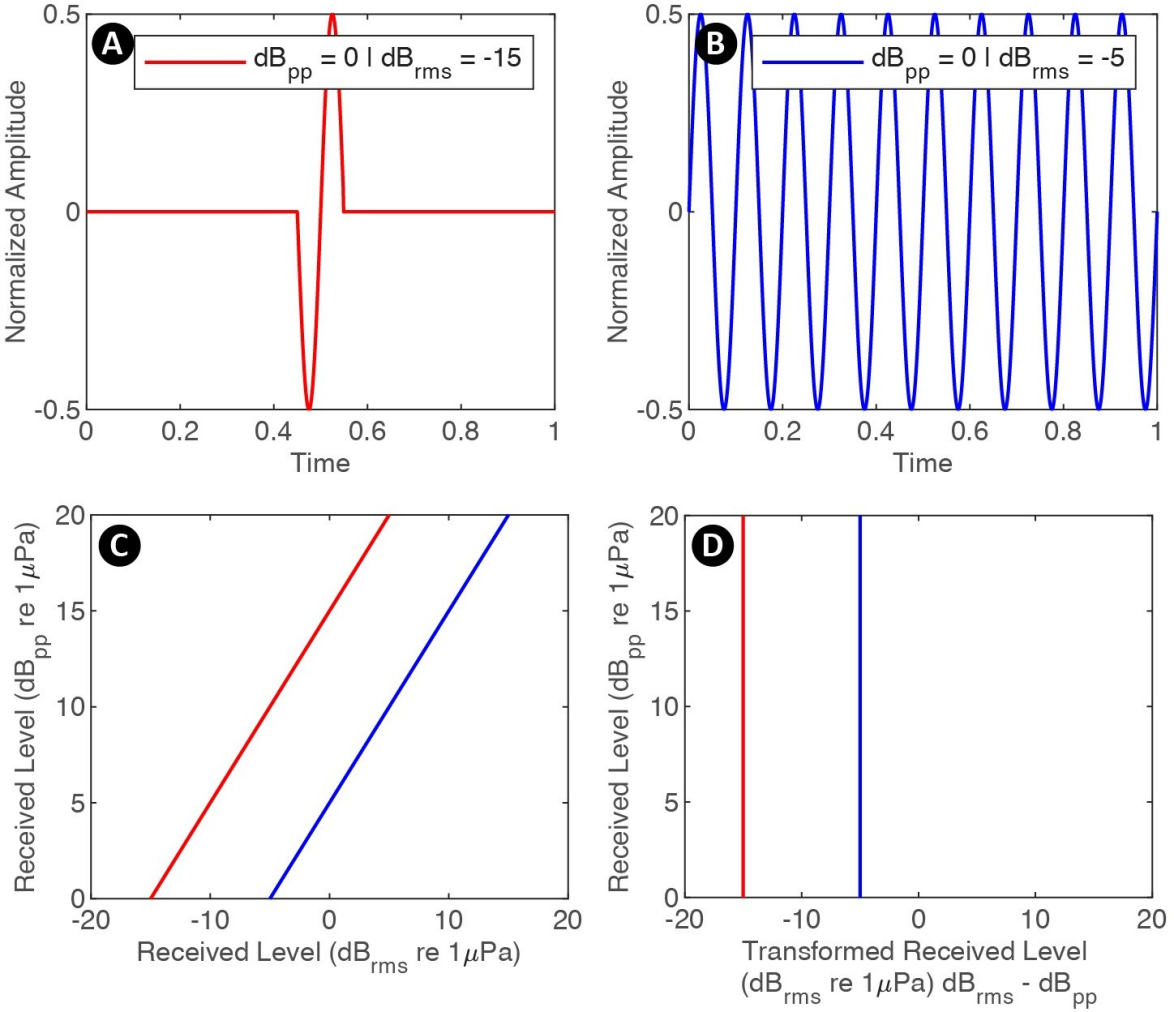
Conceptualization of transformed RL_rms_. Example signals of identical RL_pp_ with low RMS indicating a signal characterized by few high amplitude cycles (A), and high RMS indicating a sustained signal with many cycles at high amplitude (B). (C) Signal A and B with varying RL_pp_ plotted as a function of RL_pp_ versus RL_rms_ displaying a linear relationship, (D) vertical slope for transformed RL_rms_.

After building the parameter files, and specifying the input directories and display parameters in a script (see myDataSettings as an example), the user evokes the interface by calling detEdit. Predefined parameters for eleven species of odontocetes (e.g. beaked whale, dolphin, and sperm whale) are provided in initSpParams. The data are organized and displayed in bouts of detections allowing users to annotate large batches of detections (see https://github.com/MarineBioAcousticsRC/DetEdit/wiki/Getting-Started for more details). Seven panels are displayed to provide the signal detail and context needed to discriminate between different types of detections (Fig 2). The main interactive panel displays peak-to-peak received levels (RL_pp_ dB re 1μPa) of detected signals, with the concurrent LTSA, and time between detections (time difference between one detected signal and the next). The concurrent waveforms, spectra, transformed version of root-mean-square (RL_rms_ dB re 1μPa) and peak frequencies are displayed on additional interactive panels. RL_rms_ summarizes the distribution of energy within a waveform, and is transformed to facilitate the annotation of signals of consistent shape but with varying amplitude (Fig 3A and 3B). Presuming that signals of a consistent shape will increase linearly in both RL_pp_ and RL_rms_, the slope is arranged vertically, where transformed RL_rms_ = RMS–correction factor* (RL_pp_−RL_pp_ threshold) (Fig 3C and 3D). This transformation emphasizes values that deviate from the basic relationship. Generally for a vertical transformation when the increase in RL_pp_ is the same as in RL_rms_, a correction factor equal to one is appropriate. Signal types that do not meet this linear increment required a different correction factor to enable the vertical arrangement.

**Fig 4:**
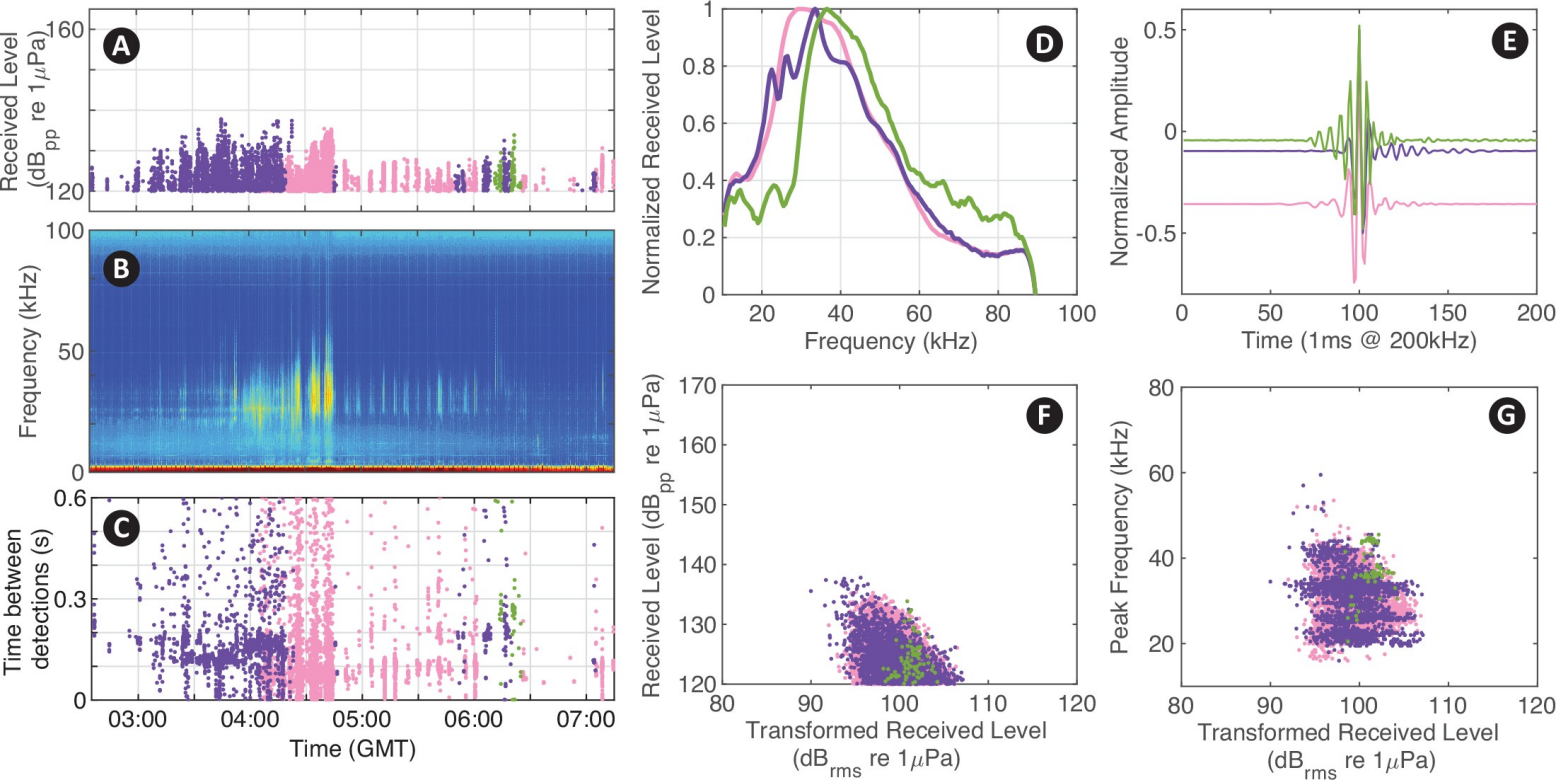
Example of labeling different detected signal types. Specified signal types of odontocete species represented in different colors, *Stenella* spp. (pink), *Grampus griseus* (purple), and *Mesoplodon europaeus* (green).

Following a simple list of keyboard shortcut commands and the use of a paintbrush tool (see https://github.com/MarineBioAcousticsRC/DetEdit/wiki/How-It-Works#Tools for more details), the user can parse the data by selecting single or multiple detections to interactively visualize the features or averaged features of the selection, and compare these parameters with the parameters of other detections within the displayed bout. Thresholds can be defined for peak frequency, transformed RL_rms_ and RL_pp_ to automate the process of annotating data (Fig 2F and 2G). Any detection lower than the selected thresholds will be automatically labeled and displayed as a false positive. All annotations can be reversed and labeled as true positives or specified signal types by using a palette of colors with the paintbrush tool (Fig 4). All changes made through the interactive interface are updated and stored in the corresponding annotation files. False detection files (FD.mat) contain all start times of signals labeled as false detections. Detection type files (ID.mat) contain all start times of signals labeled as a specified type of detection, with the corresponding color and integer value representing the species or category.

### Post-processing

After manually annotating all detections, decisions stored in different annotation file types are used to modify the detection parameters stored in TPWS files. modDet excludes all false detections and ID detections (if specified by the user) from the TPWS files and displays exploratory plots as histograms of RL_pp_, peak frequency and time interval between detections for each file. The process of using detEdit and modDet can be repeated iteratively until all detections are labeled, or a sufficiently low percentage of false detections is obtained (Fig 1). For every iteration, new detection and annotation files are generated and stored together in a common directory to keep track of all changes.

**Fig 5:**
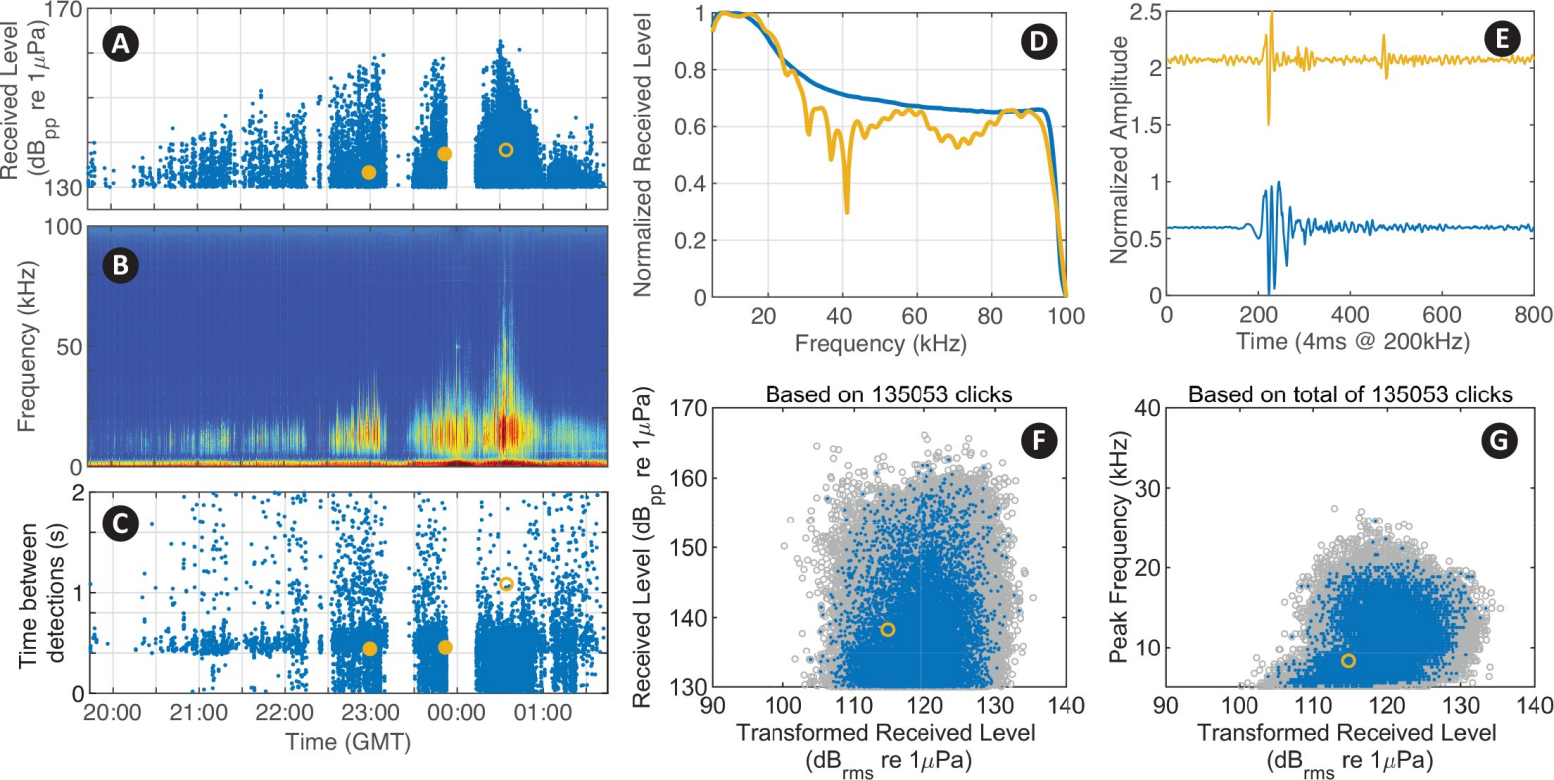
Example of evaluating false positive rates at the signal-level. Detected signals being evaluated within the bout are shown in yellow. Evaluation is done in a consecutive manner, with the current signal marked with a yellow circle, and previously evaluated signals displayed as yellow dots.

When specified using a simple keyboard shortcut commands, detEdit allows rapid estimation of a detector’s false positive rate across a systematic random sample of detections (Fig 5). The estimation procedure can be done at the signal-level where selected individual signals are sequentially evaluated as true or false within a bout. Alternatively, the procedure can be conducted at the level of a time-bin. This process consists of deciding if at least one signal within the defined interval is a true detection, whereby the entire bin is considered “true”. Signals or intervals evaluated for false positive rate estimation are selected systematically (every Nth detection) across a file. A matrix with the false positive rate per bout is built and stored in true detection files (TD.mat).

Finally, a data summary is given with histogram plots of RL_pp_, time interval between detections, and a time series of weekly presence of the true detections at the signal-level and the time-bin level (S1 Fig).

## Results

*DetEdit* has been used to analyze acoustic recordings of odontocete vocalizations [12–15]. The utility of *DetEdit* for facilitating analyses of large datasets is demonstrated with two case studies that performed species-level analyses of large acoustic datasets. A variety of reproducible results from these examples are included in the repository and described at https://github.com/MarineBioAcousticsRC/DetEdit/wiki/Getting-Started

### Interactive visualization of acoustic features to discriminate odontocete species

The acoustic features displayed in the panels of the interface are helpful parameters for distinguishing signals from multiple odontocete species. Odontocetes produce highly-directional echolocation clicks with species-specific characteristic spectral shape and duration. These characteristics can be distinguished from the panels that show averaged normalized spectral density and waveforms of all signals within bouts, and can be compared with individual selected detections.

Odontocetes often echolocate at a characteristic rate [16]. The time interval between detections (Fig 2C), also known as inter-click-interval (ICI), is most variable when multiple animals are recorded simultaneously and the received impulse trains become interleaved. When only a single animal is pointing at the sensor and echolocating, a consistent echolocation click rate is likely to be received. Since echolocation signals are directional, there may be variability in ICI even in the single animal case due to changes in behavior and the detectability associated with animal orientation. The ICI time series, together with RL_pp_ of the detections throughout the encounter, and the concurrent spectral characteristics displayed in the LTSA panel allow context-supported interpretation of the data and differentiation of signal types.

The RL_pp_ versus transformed RL_rms_ display is a unique feature of *DetEdit* that allows users to distinguish between signals with the similar maximum amplitude, but different pulse characteristics in the time domain. Signals of short duration appear on the right side of the plot and signals of long duration appear on the left side of the plot. The same transformed RL_rms_ is shown with respect to peak frequency into another display. These panels help distinguish between odontocete species’ clicks, as well as some human signals (e.g. echosounders, ship noise).

### Case Study 1: Sperm whales

A routine for verifying candidate sperm whale echolocation clicks was developed to distinguish signals with a multi-step approach (S1 Text). This case study illustrates the versatility of *DetEdit* for different labeling purposes and manual labeling of false detections. A total of 202 TB of data (containing 34 million sperm whale clicks) was verified and corresponding false positive rates were calculated.

An example of editing acoustic data with sperm whale clicks within a mixed species time period is shown in Fig 2. The window panels of both RL_pp_ and peak frequencies with respect to transformed RL_rms_ were particularly useful in distinguishing sperm whale detections from other odontocetes. In this case, the removal of dolphin clicks was possible by selecting clicks with lower RMS. To accelerate the removal of low RMS detections, a RL_pp_ threshold was implemented to automatically label all detections below the threshold as false positives (Fig 2F). Also, noisy periods of time corresponding to impulsive shipping noise were visually identified and flagged with the brushing tool for exclusion.

### Case Study 2: Delphinids

This example shows how *DetEdit* supports the identification of distinct delphinid click types within large datasets (https://github.com/MarineBioAcousticsRC/DetEdit/wiki/Getting-Started). Unsupervised clustering tools were used to automatically classify signals into categories and to assist human analysts with processing multi-species acoustic encounters [12]. A total of 171 TB of data (with 115 million dolphin clicks) was verified, and corresponding false positive rates for each species were calculated. Clusters were evaluated using the interface, with different color codes distinguishing the multiple detection types identified (Fig 4). Multiple encounters of overlapping species were visually distinguishable and flagging clicks in different colors made identification of different species possible. The selection tool supported this process by allowing manual selection of individual or multiple detections to compare with the different color-coded detections.

## Availability and future directions

*DetEdit* as a MATLAB package with example datasets for different species and detailed instructions are available from GitHub (https://github.com/MarineBioAcousticsRC/DetEdit). The current code runs under MATLAB R2014b or newer versions. More details on the implementation and the examples are given at https://github.com/MarineBioAcousticsRC/DetEdit/wiki.

Future directions include adding additional user-friendly GUI tools, incorporating clustering techniques to pre-label data using machine learning based classifiers, supporting visualization of signal templates for assisted classification, and providing certainty scores to complement classification and false positive labels.

## Supporting information

S1 Fig Example plots of data summary. (TIF)

S1 Text Detection algorithm for sperm whale echolocation clicks. (DOCX)
