# Supplementary figures and images for: DetEdit: A graphical user interface for annotating and editing events detected in long-term acoustic monitoring data
Source: PLoS Comput Biol. 2020 Jan 13;16(1):e1007598. doi: 10.1371/journal.pcbi.1007598 (PMC6980688; doi:10.1371/journal.pcbi.1007598)

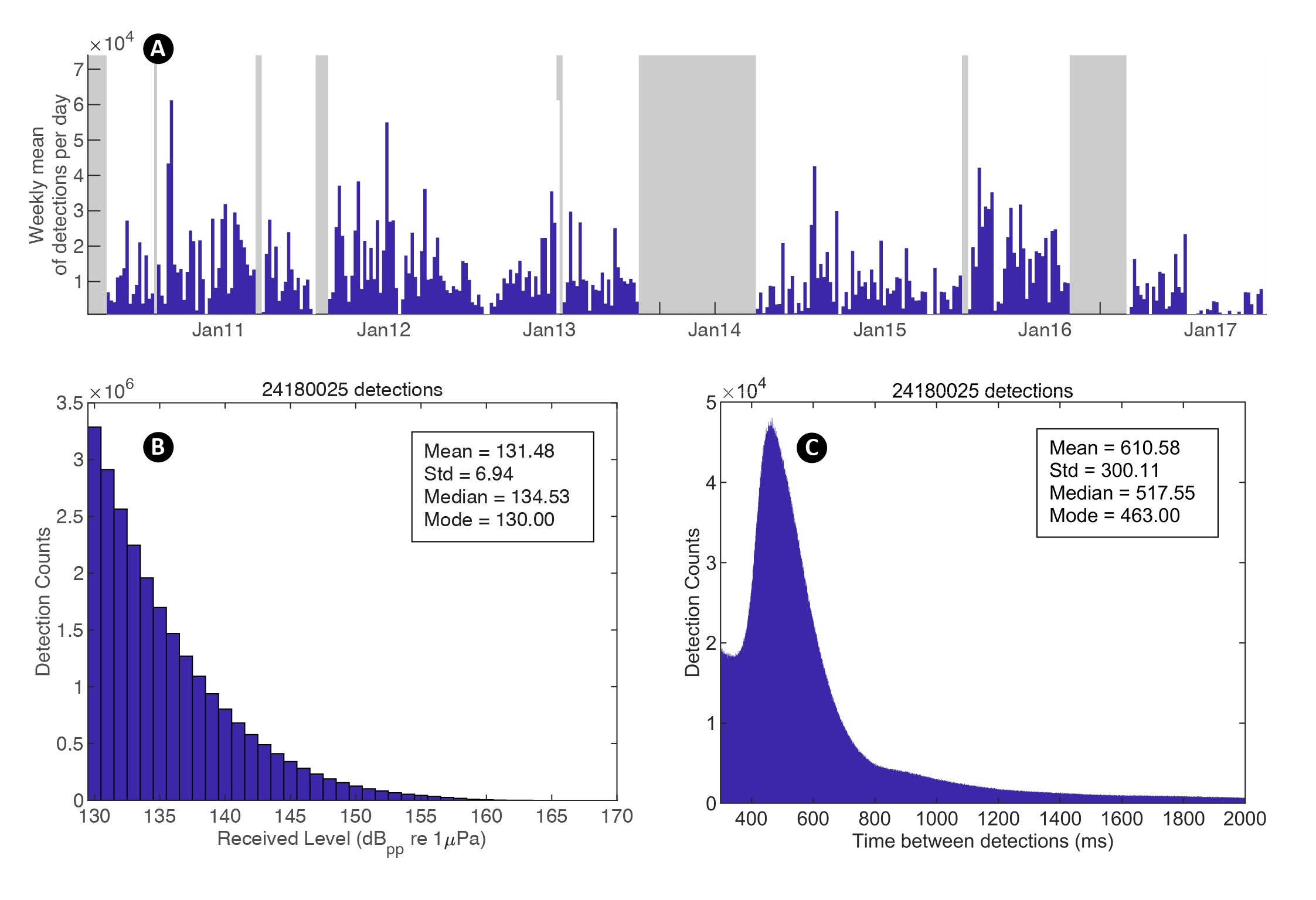

Supplement: S1 Fig — (TIF) [file pcbi.1007598.s001.tif]
