# Supplementary material for: DetEdit: A graphical user interface for annotating and editing events detected in long-term acoustic monitoring data
Source: PLoS Comput Biol. 2020 Jan 13;16(1):e1007598. doi: 10.1371/journal.pcbi.1007598 (PMC6980688; doi:10.1371/journal.pcbi.1007598)
Supplement: S1 Text — (DOCX) [file pcbi.1007598.s002.docx]

# Supporting Methods

Alba Solsona-Berga, Kaitlin E. Frasier, Simone Baumann-Pickering, Sean M. Wiggins and John A. Hildebrand

# Detection algorithm for sperm whale echolocation clicks

## Data analysis

Detection. A routine for identifying candidate sperm whale echolocation clicks implemented in MATLAB (Mathworks, Natick, MA) was developed to distinguish signals with a multi-step approach. Sperm whale wideband clicks can be easily distinguished from other marine species, but one of the difficulties is that their signals are highly similar to the impulsive cavitation sounds produced by vessel propellers. Potential click candidates were identified using a variation of a two-step approach proposed for dolphin clicks [1,2]. In the first detection step, presence of an echolocation click was determined in the spectral domain (10ms frames, 2048-point FFT, 50% overlap, Hann window) when 20 % of the frequency bins between 5 – 20 kHz exceeded a signal-to-noise ratio of -10dB threshold. The threshold was set low to allow detection of a large number of sperm whale clicks with the trade-off of many false positives, which were subsequently removed. In the second detection step, a high-resolution detector was used to return precise start and end times of individual impulses. All acoustic data were band passed (5 – 95 kHz) to minimize the influence of low-frequency noise from vessels, electrical noise, and weather. Impulses were identified within flagged times from the previous step using a Teager-Kaiser energy operator [3,4]. The noise floor was estimated at the 40^th^ percentile of the energy distribution, and Teager-Kaiser energy peaks that exceeded the noise floor by a factor of 7 were defined as potential echolocation clicks. Click start and end times were identified from each energy peak as the first and last points that were three times greater than the noise floor.

Click times that were less than 30 ms apart were merged, considering that sperm whale clicks are characterized by multiple pulses that are nearly 5 ms apart [5]. Spectra of detected signals were calculated using 4 ms of data, and a 512-point Hann window centered on the click with 50 % overlap. Spectra were corrected for the frequency-dependent hydrophone response with a transfer function. To provide a consistent detection threshold, only clicks that exceeded a received level (RL) of at least 130 dB_pp_ re 1 µPa were retained for further analyses.

Shipping passages and weather events identification. An automated classifier was developed to exclude periods of ship passages that were attributable to impulsive signals from the cavitation noise of ship propellers, and weather events. The ship impulsive signals exhibit spectral-temporal characteristics similar to those of sperm whale clicks making automatic classification challenging. Although, the elevated noise levels that dominate the ship passages made it possible to distinguish these events. The classification was used to identify ship passages from long-term spectral averages (LTSA, [6]) to remove detected impulses within the event. LTSAs are long-term spectrograms, which are created using a time average of 5 s and frequency bins of 100 Hz (calculated using Hann window without overlap). Noise produced from the high-frequency acoustic recording packages (HARP) when writing to the disk was removed by excluding the first 15 s interval of each sequential 75 s acoustic record [7]. Average power spectral density (APSD) estimates were then computed in 2-h blocks of data over three frequency bands, referred to hereafter as the low (1 – 5 kHz), medium (5 – 10 kHz), and high (10 – 50 kHz) band, and corrected for the hydrophone transfer function (Fig 1). Missing values due to the system noise removal occur regularly and interfere in the detection of events. The detector searches for events that are above a noise floor threshold by a defined duration in each frequency band. Missing values were interpolated from neighboring averages to obtain a uniform APSD and find start and end times of the events. An adaptive threshold was used to determine transient signals (e.g., odontocetes, ship passages, weather) above the background noise over the three frequency bands. Two state levels of the ASPD were estimated using a histogram method, where the histogram was divided into two equal regions, and the mode of each region was taken as the lower and upper level. The time-dependent threshold was calculated as the mean of the lower- and higher-state levels. Start and end times of high amplitude events, referred to hereafter as transients, were identified when crossing over the threshold, and only those longer than 150 s were retained.

Transients present in the 3 bands were classified as ship passages. However, cases of close encounters of odontocetes also showed transients in all three bands. These cases were identified as odontocetes only if the transient in the high band was longer than that in the medium band, and the duration of the transient in the medium band was 2/3 longer than that in the lower band. Presence of distant ships did not yield transients in the high band. Those passages were identified if duration in the low and medium band were above 250 s and differentiated from sperm whale encounters if transients in the medium band were at least 2/3 smaller than those in the lower band. Weather noise events (e.g., rain and storms) do not produce impulsive sounds. These events were not excluded from analysis and were distinguished from the ship passages by comparing RLs. RLs were taken from 1-min sliding-windows with 55 s overlap of the APSD estimates. RLs of the transients in the three bands were compared, and ship passages were defined if: RLs in the low band were above 80 dB, while below 60 dB in the medium and higher band or the differences between RLs in the low band and the mean of RLs in the medium and higher band were above 15.

To ensure that ship passages on the edges of the 2-hour analysis block window were also detected, two overlapping sliding window were implemented with the window being analyzed. The overlapping windows, each of 2-h block, started 30 min before and after the current window.. Ship presence was determined if ship passages were detected in at least two of the three overlapping windows. All identified ship passages were then manually reviewed by an experienced acoustician using the custom software program *Triton* [6] to minimize the inclusion of such events in the sperm whale dataset. Following this process, a misclassification rate below 0.004 was obtained for sperm whale encounters.

**Removal of non-target species**: A basic classification method based on spectral click shape was implemented to discard obvious non-sperm whale clicks, such as beaked whale and delphinid clicks. Often peak frequencies were used to discriminate sperm whale clicks because of their lower frequency compared to other odontocetes. When animals were very close to the acoustic recorder clicks often had more energy in the higher frequencies, making it more difficult to differentiate them with confidence. Clicks were only removed from the analysis if they had a peak frequency above 50 kHz or a second peak above 22 kHz. Sperm whales have a distinctive spectral shape with most energy in frequencies below 30 kHz, while delphinids and beaked whales have more energy in higher frequencies; however, spectral shapes for dolphins and beaked whales are expected to have more variation because of the effect of acoustic attenuation for high frequencies. To characterize the spectral shape of each click, the difference across spectral levels of a specified frequency band was computed as

$$\Delta u= {mean(u}_{high})- {mean(u}_{low})$$

where *u*_high_ is the vector of spectral levels of the click across the frequency range of 35 – 45 kHz, and *u*_low_ is the vector of spectral levels from 10 – 20 kHz. Normalized click spectral levels (*u*_n_) between [0, 1] were computed as

$$u_{n}= \frac{u-min(u)}{max(u-min(u))}$$

where *u* is the vector of spectral levels of one click across the frequency range. Signals with a spectral difference (Δ*u*) above -5 and a median of the normalized spectra (*u*_n_) above 0.8 were defined as high-frequency spectral shapes, which were linked to beaked whale and delphinid clicks. If more than 85 % of the clicks were discarded in the audio file, the section was linked to a specific anticipated false positive source (e.g. ship noise, seismic surveys, delphinid, and beaked whale) and was discarded from further analysis. Isolated clicks separated from neighboring signals by more than 5 seconds were also discarded.


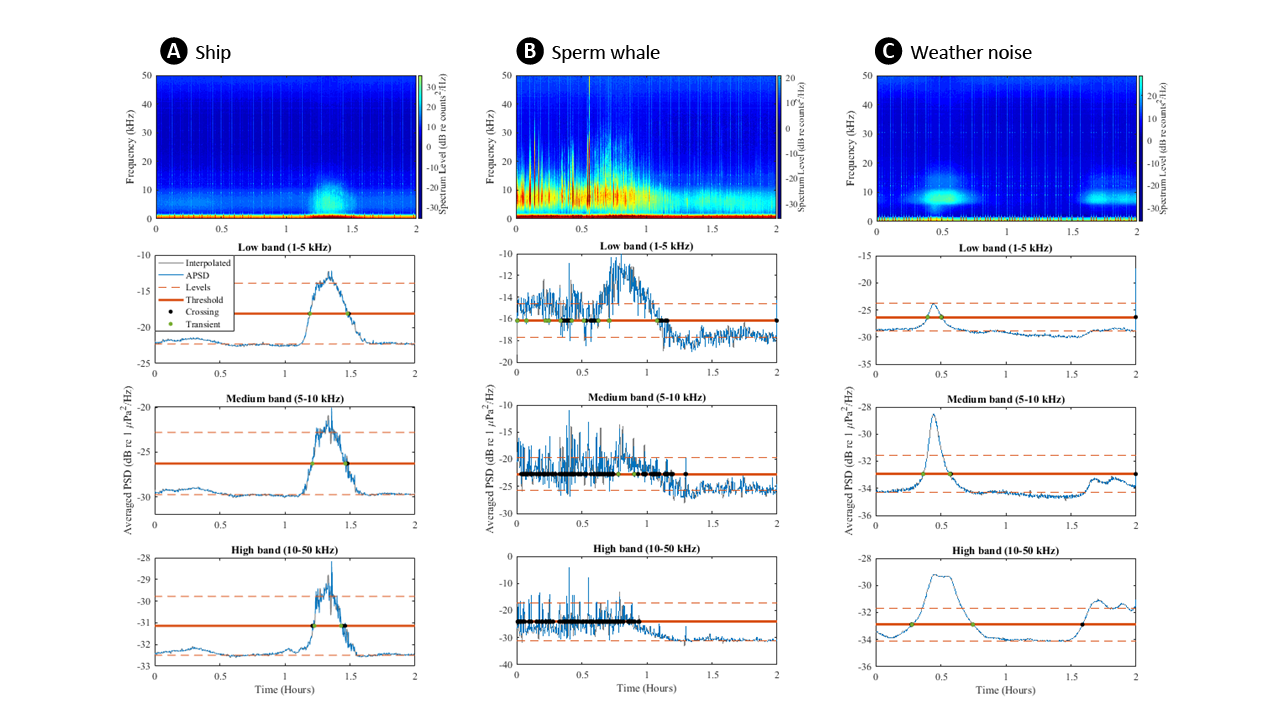


Figure 1. Event types including (A) a ship passage, (B) sperm whale encounter, and (C) weather noise in 2-h LTSAs (upper panels). LTSAs are 2000 samples (100 Hz bins) with 50% overlap and color represents sound pressure spectrum level. Concurrent averaged power spectral densities (APSD) for the three frequency bands (lower panels) with transients identified if duration between start and end crossing points of the event was above 150 s.

# References

1. Roch MA, Klinck H, Baumann-Pickering S, Mellinger DK, Qui S, Soldevilla MS, et al. Classification of echolocation clicks from odontocetes in the Southern California Bight. J Acoust Soc Am. 2011;129: 467–75. doi:10.1121/1.3514383

2. Soldevilla MS, Henderson EE, Campbell GS, Wiggins SM, Hildebrand JA, Roch MA. Classification of Risso’s and Pacific white-sided dolphins using spectral properties of echolocation clicks. J Acoust Soc Am. 2008;124: 609–624. doi:10.1121/1.2932059

3. Kaiser JF. On a simple algorithm to calculate the “energy” of a signal. International Conference on Acoustics, Speech, and Signal Processing. IEEE; 1990. pp. 381–384. doi:10.1109/ICASSP.1990.115702

4. Kandia V, Stylianou Y. Detection of sperm whale clicks based on the Teager-Kaiser energy operator. Appl Acoust. 2006;67: 1144–1163. doi:10.1016/j.apacoust.2006.05.007

5. Zimmer WMX, Madsen PT, Teloni V, Tyack. MPJ and PL. Off-axis effects on the multipulse structure of sperm whale usual clicks with implications for sound production. J Acoust Soc Am 118(5)3337-3345 2005. 2005;118: 9. doi:10.1121/1.2082707

6. Wiggins SM, Hildebrand JA. High-frequency Acoustic Recording Package (HARP) for broad-band, long-term marine mammal monitoring. International Symposium on Underwater Technology 2007 and International Workshop on Scientific Use of Submarine Cables & Related Technologies 2007. Institute of Electrical and Electrongics Engineers, Tokyo, Japan; 2007. pp. 551–557.

7. Wiggins SM, Hall JM, Thayre BJ, Hildebrand JA. Gulf of Mexico low-frequency ocean soundscape impacted by airguns. J Acoust Soc Am. Acoustical Society of America; 2016;140: 176–183. doi:10.1121/1.4955300
